# Mucosal IgG in inflammatory bowel disease – a question of (sub)class?

**DOI:** 10.1080/19490976.2019.1651596

**Published:** 2019-09-03

**Authors:** Tomas Castro-Dopico, Menna R. Clatworthy

**Affiliations:** a Molecular Immunity Unit, Department of Medicine, University of Cambridge, Cambridge, UK; b NIHR Cambridge Biomedical Research Centre, Cambridge, UK; c Cellular Genetics, Wellcome Trust Sanger Institute, Hinxton, UK

**Keywords:** IgG, Fcγ receptors, inflammatory bowel disease, ulcerative colitis, Crohn’s disease, subclasses

## Abstract

Immunoglobulins (Igs) form a cornerstone of mucosal immunity. In the gastrointestinal tract, secretory IgA and IgM bind to commensal microorganisms within the intestinal lumen to prevent them from breaching the intestinal epithelium – a process known as immune exclusion. In recent years, there has been renewed interest in the role of IgG in intestinal immunity, driven in part by a genetic association of an affinity-lowering variant of an IgG receptor, FcγRIIA, with protection from ulcerative colitis (UC), a subclass of inflammatory bowel disease (IBD). We recently demonstrated a role for IgG and Fcγ receptor signalling in driving pathogenic IL-1β production by colonic mononuclear phagocytes and the subsequent induction of a local type 17 response in UC. Here, we discuss the potential relevance of our observations to the other major subclass of IBD – Crohn’s disease (CD) – where the genetic association with *FCGR* variants is less robust and consider how this may impact therapeutic interventions in these disease subsets.

## Introduction

Secretory immunoglobulin (IgA) and IgM antibodies play a fundamental role in maintaining mutualism between the host and the microorganisms that colonise our mucosal surfaces.^1^ At these sites, IgA limits microbial penetrance, promotes bacterial elimination, and educates the mucosal immune system through antigen capture.^2–4^ However, IgG has also been described in the healthy human and murine gut.^5^ Maternal IgG plays a key role in neonatal intestinal immunity where it provides mucosal protection and immune education prior to the development of post-natal immunity.^6–8^ However, intestinal IgG responses are limited in the healthy adult gut, given the potential pro-inflammatory effects of this antibody isotype in complement fixation and immune cell activation via Fcγ receptor (FcγR) engagement.

In the context of intestinal infection, several studies have identified a critical role for B cells and IgG in pathogen containment and elimination – dominant effects that supersede the involvement of IgA and IgM.^9–13^ For example, IgG is required for neutralization of *Citrobacter rodentium* in mice, a model of attaching-effacing enteropathogenic *Escherichia coli* infection in humans. This protection can be passively transferred to offspring *in utero* and through breast milk.^9,11^ Therefore, mucosal immunity retains the capacity to elicit potent IgG-mediated inflammatory responses if required to promote pathogen clearance.

Historical studies have identified an increase in mucosal IgG responses in patients with inflammatory bowel disease (IBD), a chronic relapsing inflammatory disease of the gastrointestinal (GI) tract.^14,15^ Indeed, intestinal IgG^+^ cells and circulating anti-commensal IgG have been observed in both major subclasses of IBD, Crohn’s disease (CD) and ulcerative colitis (UC). However, the functional consequences of this response remained largely unknown, given the long-standing belief that B cells do not participate in IBD pathogenesis. Our recent study sheds light on the mechanisms by which mucosal IgG drive intestinal inflammation in UC, through the engagement of FcγRs on local macrophages, leading to subsequent type 17 T cell activation. However, whether this represents a universal pathogenic mechanism across IBD subsets is unclear, particularly considering the differing strength of genetic association between *FCGR* variants and UC and CD susceptibility.

## Inflammatory bowel disease

### Immunopathology and genetics

IBD is divided into two major subclasses, CD and UC, that differ in their clinicopathological phenotype.^16^ CD can involve any part of the intestine from mouth to anus and is characterised by transmural inflammation, whereas UC is confined to the large intestine and inflammation is typically limited to the mucosa. At the core of disease susceptibility is an aberrant immune response towards the microbiota, precipitated by poorly defined environmental and genetic factors. Intestinal epithelial, stromal, and myeloid cells produce key inflammatory mediators, including tumor necrosis factor (TNF) and interleukin (IL)-23, while adaptive immunity is skewed towards mixed Th1/Th17 and Th2/Th17 responses in CD and UC, respectively. Unsurprisingly, immunosuppressive agents form the mainstay in IBD therapy, with monoclonal antibodies that target some of these cytokines, particularly TNF and IL-23, showing success in the clinic.^17–19^

**Figure 1.**
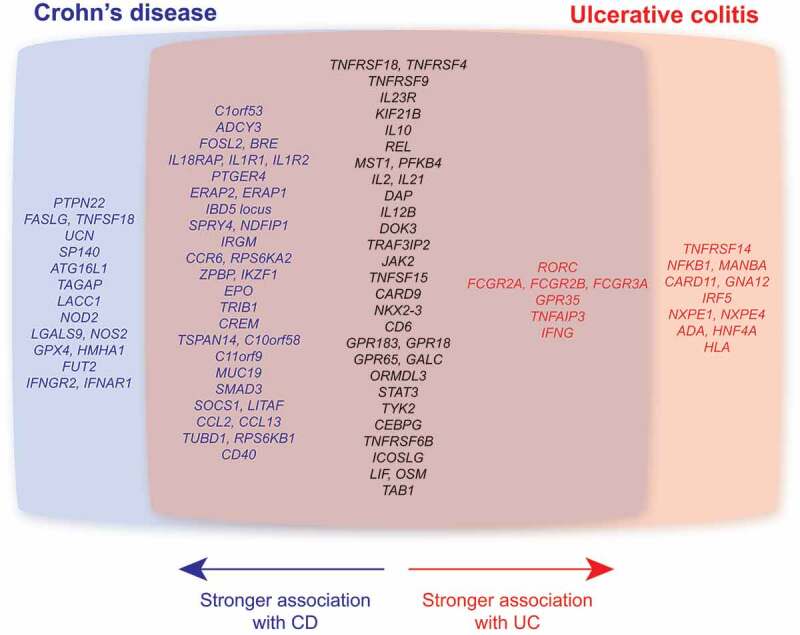
**Major genetic associations in inflammatory bowel disease**. Venn diagram of major genetic associations in IBD (candidate genes from single nucleotide polymorphisms (SNPs) with P value <1 × 10^−13^.^20^ Common genes are subdivided based on greater association with CD (blue) or UC (red).

While CD and UC share several genetic associations, including components of the IL-23-Th17 axis, there are also major genetic determinants of disease susceptibility that are unique to each IBD subclass (Figure 1).^20^ UC susceptibility is linked to genes involved in epithelial barrier function and the major histocompatibility complex region, near HLA class II genes. In contrast, CD is linked to defects in microbial sensing. NOD2 mutation homozygotes exhibit ~20-fold increased risk for CD,^21^ while a further susceptibility locus maps to the autophagy gene *ATG16L1*, a process required for growth restriction of some intracellular bacteria, such as *Salmonella typhimurium*.^22^ Indeed, several IBD susceptibility loci map to genes involved in primary immunodeficiencies (PIDs), disorders characterised by severe susceptibility to infection, including *CARD9, IFNGR2* and *STAT3*, particularly in CD.^20^

### Fcγ receptors in IBD

The potential involvement of IgG and FcγRs in IBD pathogenesis was highlighted by the consistent identification of an activating Fcγ receptor gene variant, *FCGR2A-*R/H131, among associated risk loci in UC across genome wide association (GWA) studies in multiple populations.^20,23^ Specifically, the *FCGR2A* variant rs1801274 encoding a receptor with low affinity for IgG (R131) is associated with protection from UC,^23^ suggesting a pathogenic role for IgG. FcγRs are cell surface receptors widely expressed by innate immune cells and B cells that bind to the Fc domain of IgG antibodies.^24^ There are several activating FcγRs in both humans (FcγRI, FcγRIIA, FcγRIIIA, and FcγRIIIB) and mice (FcγRI, FcγRIII, and FcγRIV), whose crosslinking by IgG immune complexes or opsonized cells leads to phosphorylation of immunoreceptor tyrosine based activating motifs (ITAMs) located on the intracellular domain or on the associated common γ-chain, leading to cellular activation.^25^ There is also a single inhibitory receptor in both humans and mice, FcγRIIB, that contains an intracellular immunoreceptor tyrosine-based inhibitory motif (ITIM) that can recruit phosphatases to signalling synapses to dampen IgG-mediated activation signalling.

In our recent study, we demonstrated a significant enrichment of IgG-opsonized commensals in stool samples from patients with UC that positively correlated with disease severity.^26^ Transcriptomic analysis of human colonic UC biopsies demonstrated an enrichment of genes associated with activating *FCGR* signalling, and implicated an immune cell network, at the heart of which was IL-1β-producing intestinal FcγRIIA-expressing mononuclear phagocytes (MNPs). Having demonstrated FcγRIIA-dependent IL-1β production by human macrophages in response to IgG *in vitro*, we validated a functional effect of this IgG-macrophage axis *in vivo* by using a mouse model of intestinal inflammation – dextran sodium sulfate (DSS)-induced colitis. In this setting, manipulation of the intestinal MNP FcγR signal strength, using transgenic mouse strains with varying inhibitory FcγR expression, regulated colitis severity. Exacerbated FcγR signalling increased colonic MNP IL-1β production and subsequent neutrophil infiltration and local type 17 immunity. In contrast, MNP-specific overexpression of FcγRIIB reversed this susceptibility, directly linking the FcγR-MNP axis to disease progression. Furthermore, it is noteworthy that elevated FcγRIIA expression was linked to infliximab-resistant UC, suggesting this network may also contribute significantly to treatment-refractory UC.

## Immunoglobulin G in Crohn’s disease and ulcerative colitis

**Figure 2.**
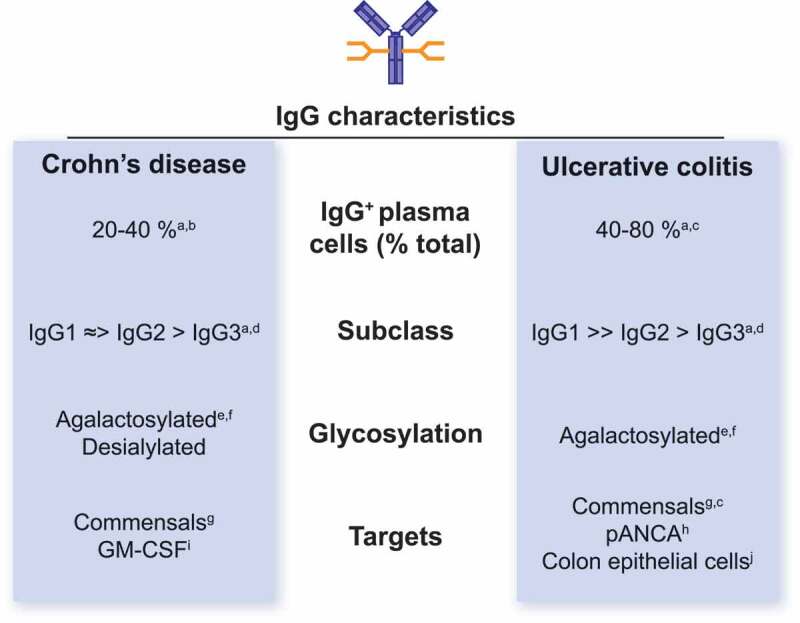
**IgG characteristics in Crohn’s disease and ulcerative colitis**. Summary of the differences in IgG responses between CD and UC patients, including frequency of plasma cell pool, IgG subclass distribution, glycosylation status, and antigen specificity. pANCA = perinuclear anti-neutrophil cytoplasmic antibodies; GM-CSF = granulocyte macrophage-colony stimulating factor.

Our study focused on the pathogenesis of UC, given its stronger genetic link to FcγRIIA variants (CD *P* value = 1.53 x 10^−7^; UC *P* value = 2.12 x 10^−38^)^20^ (Figure 1). Whether similar immune pathways contribute to CD pathogenesis is unclear. It is noteworthy that although potent mucosal IgG responses are also observed in CD, subsequent studies have demonstrated significant heterogeneity both between CD and UC and within IBD subclasses in the IgG response, including differences in IgG frequency, subclass distribution, antigen specificity, and glycosylation state (Figure 2).^14,27–32^ Here we discuss some of these differences and how they may be affected by the genetic context of each IBD subclass.

### IgG subclasses

Although there is a strong genetic association of *FCGR2A* variants with UC, the link with CD is weaker, despite the fact that mucosal IgG is increased to a similar extent in CD and correlates with disease severity.^33–35^ Asano *et al*. found no association of the *FCGR2A* SNP with CD, a finding confirmed by some GWA studies,^36^ however, some candidate gene studies have also implicated rs1801274 as protective in CD,^37^ as did a large meta-analysis of GWA studies.^20^ A small candidate gene study also identified an association between FCGR3A*A559C (rs396991, encoding a valine for a phenylalanine at amino acid 158 in the extracellular domain of the receptor, (FcγRIIIA-F158V)) and CD,^38^ but this has not been confirmed in larger GWA studies.

One potential explanation for the differing associations of UC and CD with *FCGR* genotypes may lie in the differences observed in IgG subclass distribution and in the mucosal plasma cell pool between the two IBD subclasses. In UC patient biopsies, up to 80% of mucosal plasma cells express IgG, the majority being IgG1^+^ – an inflammatory IgG subclass capable of potent activating FcγR engagement and complement activation.^39–42^ In contrast, IgG^+^ cells make up a smaller fraction of the total mucosal plasma cell pool in CD, with a greater contribution from IgM.^33,34,39^ Importantly, IgG^+^ plasma cells in CD are equally distributed between IgG1 and IgG2 subclasses. IgG2 is known to have reduced inflammatory capacity relative to IgG1, making up a large proportion of the mucosal IgG pool in health.^32^ Smaller populations of IgG3^+^ cells have been observed in both IBD subclasses, the IgG subclass with most potent complement-fixing and FcγR signalling capacity.^42^ Furthermore, these studies highlight large inter-individual variability in the magnitude of mucosal IgG that strongly correlates with disease severity, a finding corroborated by ourselves in UC by measuring luminal commensal-bound IgG levels.^26^ Therefore, IgG appears to associate with both subclasses of IBD, but the level and inflammatory subclass frequency is highest in UC.

### Glycosylation

IgG heavy chains are post-translationally modified by glycosylation at asparagine 297 within the Fc region – a modification critically required for effector function – with over 900 glycoforms possible.^40,43^ Unsurprisingly, specific glycoforms can variably and profoundly alter IgG effector function; for example, de-fucosylation increases the binding affinity of IgG for activating FcγRIIIA (but not FcγRIIB) 10–50 fold.^44^ Abnormalities in the IgG glycome have been observed in several autoimmune and inflammatory diseases, including IBD, and following infection.^45^

Severity-dependent agalactosylated IgG, associated with enhanced complement activation, has also been observed in IBD patients, is highest in CD and predictive of poor prognosis in UC.^46–48^ CD patients further exhibit a reduction in IgG sialylation, a modification that promotes anti-inflammatory IgG activity via reduced activating FcγR binding. The specific functional effects of these different IgG glycoforms in IBD patients, however, is unknown. Indeed, a systematic analysis of IgG subclasses and glycosylation state in CD and UC, with parallel information on site-specific disease, disease severity, and treatment response, remains to be performed.

### Antigen specificity

As well as differences in the pro-inflammatory potential of IgG between CD and UC patients, differences in antibody specificity between IBD subclasses have also been highlighted. Generally, bone fide commensal reactivity appears to be more widespread in CD.^28^ Mucosal IgG exhibits greater reactivity towards non-pathogenic commensal strains and microbial antigens, including flagellin and *Saccharomyces cerevisiae* mannan (ASCA), with little cross-reactivity with non-intestinal bacterial or viral species.^27,30,49–51^ In contrast, the development of autoantibodies is more prominent in UC. Circulating antibodies against colon epithelial goblet cells have been identified,^29^ while perinuclear anti-neutrophil cytoplasmic antibodies (pANCA) are observed in the majority of active UC patients.^52,53^ Although autoantibodies are the exception rather than the rule in CD patients, anti-GM-CSF antibodies have been described, are associated with severe disease, and may also contribute to the impaired anti-microbial responses by neutrophils and macrophages in the gut.^54,55^

Bacterial sequencing of IBD stool samples has yielded remarkable insights into the species targeted by IgA, allowing for functional validation of key commensal and pathobiont species, such as *Clostridiales* and *Ruminococcaceae* species in CD and *Eubacterium dolichum* in UC.^56,57^ Future studies will help to resolve microbial species targeted by IgG in IBD patients across disease subclasses.

### IgG-genetic interactions

**Figure 3.**
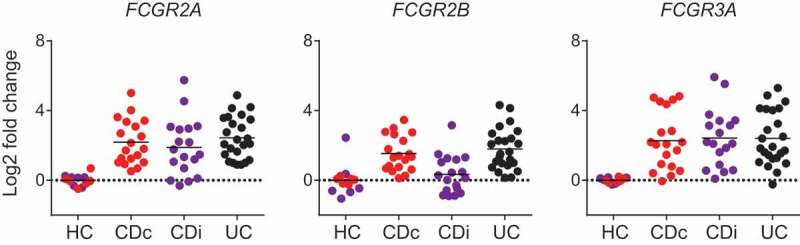
**FcγR expression in IBD mucosal biopsies**. Transcriptomic analysis of FCGR gene expression in mucosal biopsies from UC (n = 24), colonic CD (CDc; n = 19), and ileal CD (CDi; n = 18) patients and healthy controls (ileum n = 6; colon n = 6). Data generated from GEO: GSE16879.

While UC and CD share numerous genetic associations, several major genetic determinants of susceptibility are unique to each subclass (Figure 1). It may therefore be that these differing susceptibility pathways influence the overall role of IgG and FcγR in disease pathogenesis or progression in different ways. Therefore, despite the presence of anti-commensal IgG in CD, and similar levels of FcγR transcript enrichment in CD biopsies relative to healthy controls as observed in UC (Figure 3), the functional effect is dissimilar because of interactions with other pathogenic pathways at play.

As noted previously, CD is linked to defects in intracellular microbial sensing. Therefore it is of relevance that the FcγRIIA-R131 polymorphism that mediates protection in UC has been associated with susceptibility to a number of infections, including invasive pneumococcal disease,^58,59^ and that IgG and FcγRs play a functional role in defense against mycobacteria, intracellular pathogens.^60^ Therefore, the deleterious effects of FcγRIIA-R131 on bacterial clearance might outweigh its beneficial, anti-inflammatory effects in CD.

In mice, B cells play a critical role in the clearance of *Citrobacter rodentium*, an attaching-effacing enteropathic bacterium widely used to model human IBD pathology. This protection is dually dependent on IgG-FcγR signalling and CD4^+^ T cells, but independent of secretory IgA.^10,61,62^ It is noteworthy that *Nod2*-deficient mice, which are susceptible to *C. rodentium* challenge, exhibit an impaired local anti-microbial IgG response, suggesting local IgG dysfunction may compound invasive bacterial dissemination that precipitates colitis.^63^ In addition, although anti-microbial IgG is elevated in CD patients, the quality and nature of this response is likely to vary according to individual genetic and environmental factors.

Secondary considerations regarding this differential association arise from the inflammatory networks contributing to CD and UC pathogenesis.^64^ While the IL-23-Th17 axis is central to both IBD subclasses, lamina propria cells isolated from CD and UC patients produce excessive levels of IFNγ and IL-13, respectively.^64,65^ In turn, this may impact the consequences of FcγR signalling by intestinal immune cell subsets. For example, IFNγ-programmed M1-like macrophages are unaffected by dual-stimulation with TLR and FcγR ligands relative to TLR stimulation alone, while M2-like macrophages and DCs exhibit a potent Th17-inducing phenotype in response to IgG-opsonised bacteria.^66,67^ Whether this phenotype translates to intestinal macrophages isolated IBD patients, however, remains to be investigated.

## Conclusion

While mucosal IgG responses are common to both UC and CD, significant differences are observed in the targets and inflammatory characteristics of these responses that may impact disease susceptibility. Future studies will be needed to dissect the biological consequences of this heterogeneity *in vivo* and how IgG-targeted therapeutics may be used to greatest effect. Given its role in pathogen defense, FcγR blockade may be detrimental in CD by promoting microbial penetrance and dissemination in patients compounded by genetic variants in PRRs or autophagy-related pathways. Conversely, IgG blockade may be a particularly attractive target in severe IBD patients refractory to conventional therapies, where similar interventions, such as intravenous Ig, have shown potential efficacy in the past.

## Methods

Publicly available microarray datasets were downloaded from GEO (https://www.ncbi.nlm.nih.gov/geo/) along with appropriate chip annotation data. All analyses were carried out using R. All datasets were downloaded as raw intensity matrices. Data was normalized using RMA and limma. Probes were reduced to one probe per gene by selecting the probe with the greatest variance across the samples using the gene filter package.
